# Structural basis of substrate recognition by a novel thermostable (*S*)-enantioselective ω-transaminase from *Thermomicrobium roseum*

**DOI:** 10.1038/s41598-019-43490-2

**Published:** 2019-05-06

**Authors:** Sunghark Kwon, Jun Hyuck Lee, Chang Min Kim, Hyunseok Jang, Hyungdon Yun, Ju-Hong Jeon, Insuk So, Hyun Ho Park

**Affiliations:** 1 College of Pharmacy, Chung-Ang University, Dongjak-gu, Seoul 06974 Republic of Korea; 2 Unit of Polar Genomics, Korea Polar Research Institute, Incheon, 21990 Republic of Korea; 3 Department of Systems Biotechnology, Konkuk University, Seoul, 05029 Republic of Korea; 4 Department of Physiology and Biomedical Sciences, Institute of Human-Environment Interface Biology, Seoul National University College of Medicine, Jongno-gu, Seoul 03080 Republic of Korea

**Keywords:** X-ray crystallography, Enzyme mechanisms

## Abstract

Transaminases catalyze the reversible transfer reaction of an amino group between a primary amine and an α-keto acid, utilizing pyridoxal 5′-phosphate as a cofactor. ω-transaminases (ωTAs) recognize an amino group linked to a non-α carbon of amine substrates. Recently, a novel (*S*)-enantioselective ωTA from *Thermomicrobium roseum* (Tr-ωTA) was identified and its enzymatic activity reported. However, the detailed mechanism of (*S*)-enantioselective substrate recognition remained unclear. In this study, we determined the crystal structure of Tr-ωTA at 1.8 Å resolution to elucidate the mechanism underlying Tr-ωTA substrate (*S*)-enantioselectivity. A structural analysis of Tr-ωTA along with molecular docking simulations revealed that two pockets at the active site tightly restrict the size and orientation of functional groups of substrate candidates. Based on the structural information and docking simulation results, we propose a comprehensive catalytic mechanism of Tr-ωTA. The present study thus provides structural and functional insights into the (*S*)-enantioselectivity of Tr-ωTA.

## Introduction

Transaminases reversibly catalyze the transfer reaction of an amino group from an amine donor to a carbonyl acceptor, mediated by pyridoxal 5′-phosphate (PLP) as a cofactor. These enzymes are classified by diverse criteria including fold type (I and IV), sequence pattern (I–IV), and amino group position of the donor substrate (α, β, γ, and ω)^1,2^. α-, β-, and γ-transaminases recognize substrates containing an amino group bound to α, β, and γ carbon atoms, respectively^2^. ω-transaminases (ωTAs), in contrast, constitute the comprehensive enzymes that transfer an amino group bound to a non-α carbon of amine substrates^1,2^. ωTAs have gained considerable attention owing to their enantioselectivity, which is considered as a key property in the field of pharmaceutical chemistry and the fine chemical industry^3–5^. Industrial applications of ωTAs have been attained through both kinetic resolution and asymmetric synthesis. For kinetic resolution, undesired enantiomers comprising half of the produced racemic compounds are converted to ketone products, leaving the remaining, preferred enantiomers in their native form^3^. For asymmetric synthesis, preferred ketone compounds accept an amino group, yielding the desired chiral amines^3^.

This unique enzymatic property of ωTAs has led to active structural studies regarding the enantioselectivity of ωTAs. The active site geometry formed with PLP has been considered as a determinant factor for the enantioselectivity^6–8^. The amino group transfer accompanying PLP comprises two consecutive half reactions^6,7^, with the first being the oxidative deamination process of a primary amine. The second half reaction, in contrast, proceeds via the reductive amination process of a ketone compound. PLP is involved in both reactions as a mediator of the amino group. The amination reaction is initiated by substituting the amino group from an amine donor for the ε-amino group of a Lys residue covalently bound to the C4′ atom on PLP, resulting in the formation of pyridoxamine-5′-phosphate (PMP). The amino group of PMP is transferred to a ketone compound as an amino group acceptor via the second half reaction. In both reactions, PLP/PMP is located in the PLP/PMP binding site through coordination with adjacent residues. The spatial position of PLP primarily determines the enantioselectivity peculiar to each ωTA by favorably orienting toward the substrate entry direction between the *Re*- and *Si*-faces^9^. Previous structural studies have revealed that ωTAs have two pockets (the P- and O-pockets) in the active site^2,5,10^. The P-pocket is located near the phosphate group of PLP, whereas the O-pocket is positioned in the proximity of the O3′ atom on PLP. The two pockets differ from each other in size, depending on the class of ωTAs^5,8^. The resulting volumetric difference renders the substrate recognition more stringent regarding enantioselectivity^5,8,10^.

ωTAs belonging to fold type I and class III exhibit (*S*)-enantioselectivity^2^. Several (*S*)-enantioselective ωTAs have been identified on the basis of sequence and biochemical analyses^11–19^ along with structural studies^10,20–23^. An (*S*)- enantioselective ωTA from *Vibrio fluvialis* (Vf-ωTA) has also been engineered by incorporating unnatural amino acids, thereby improving its thermostability and resistance to organic solvents^24^. Recently, the structures of Vf-ωTA were determined with its apo^25^ and holo^26^ forms. Based on the sequence information of Vf-ωTA, a novel thermostable (*S*)-specific transaminase from *Thermomicrobium roseum* (Tr-ωTA) was also identified that exhibits diverse substrate specificity and thermostability^27^. This previous study revealed that Tr-ωTA demonstrates (*S*)-enantioselectivity and different enzymatic activities according to the size and physicochemical properties of its functional groups^27^.

However, the molecular mechanism through which Tr-ωTA possesses (*S*)-enantioselective substrate specificity is not fully understood. It also remains elusive how Tr-ωTA can recognize diverse substrates as an amino group donor. Moreover, although many ωTAs exploit pyruvate as an amino group acceptor, little is known regarding the spatial position and interaction at the active site during catalysis. These issues require detailed explanations at the molecular level. Thus, it is necessary to elucidate the structure of Tr-ωTA and the mechanism of binding to its substrates.

Herein, we report the crystal structure of Tr-ωTA at 1.8 Å resolution to clarify the substrate recognition mechanism of (*S*)-enantioselective Tr-ωTA. Along with structural analyses, molecular docking simulations were performed with various substrate candidates. The structural analysis revealed that two pockets at the active site of Tr-ωTA are formed with evolutionarily conserved residues. The docking simulations suggested that key residues at the active site are spatially arranged to recognize the functional groups of each substrate compound. Our results, thus, provide structural insight into the substrate recognition mechanism of (*S*)-enantioselective Tr-ωTA.

## Results and Discussion

### Overall structure of Tr-ωTA

**Figure 1:**
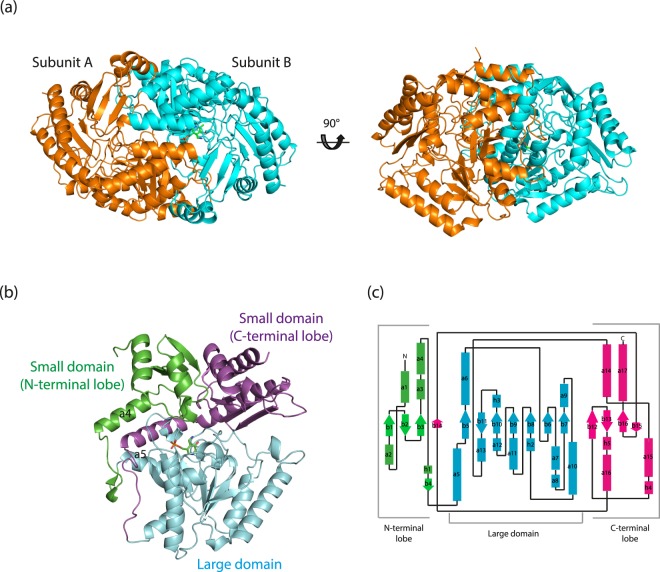
Overall structure of Tr-ωTA. (**a**) Overall structure of Tr-ωTA. The structure as a dimer form is depicted as a cartoon and viewed from two different directions. Subunits A and B are coloured orange and cyan, respectively. The PMP molecule in subunit B is represented as green sticks. (**b**) The two domains of Tr-ωTA (subunit B): large domain (residues 94–315; cyan), small domain (residues 1–93 and 316–451). The small domain is divided into two lobes (N- and C-terminal lobes): N-terminal lobe (residues 1–93; green) and C-terminal lobe (residues 316–451; magenta). (**c**) Topology diagram of Tr-ωTA. Helices and β-strands are depicted as rectangles and arrows, respectively.

The structure of Tr-ωTA was determined in the PMP-bound form at 1.8 Å resolution. Data collection and refinement statistics for Tr-ωTA are presented in Table 1. The overall structure constitutes a homodimer, with each subunit comprising a large and a small domain (Fig. 1). The large domain (residues 94–315) contains a PMP cofactor and consists of eleven helices and seven strands, adopting an α-β-α sandwich fold (Fig. 1b,c). The central β-strands form a twisted β-sheet, in which six strands, except the β11 strand, run parallel to each other (Fig. 1b,c). Two of the eleven helices comprise 3_10_-helices, of which the η3 helix contains the Lys289 residue involved in the catalytic reaction. The small domain can be divided into two lobes (N- and C-terminal lobes), which contain the corresponding terminal regions (Fig. 1b,c). The N-terminal lobe is comprised of an antiparallel β-sheet and four helices. Notably, the β-sheet is flanked by the C-terminal lobe and the α4 helix runs perpendicular across the β-sheet. The region between α4 and α5 interacts with the other subunit, playing a crucial role in forming the active site of the one subunit. The C-terminal lobe also contains an antiparallel β-sheet formed by four strands. A notable feature of the C-terminal lobe is that the α14 helix and a relatively long linker connected to the large domain are located away from the rest of the cluster of the C-terminal lobe (Fig. 1b), with the linker being responsible for forming the PLP-binding site in the other subunit.

**Table 1:** Data collection and refinement statistics for Tr-ωTA.

**Data collection**	
Space group	*R*3
Unit cell parameter *a*, *b*, *c* (Å)	
*a*, *b*, *c* (Å)	*a* = *b* = 118.3, *c* = 170.5
α, β, γ (°)	α = β = 90, γ = 120
Resolution range (Å)^a^	50.00–1.80 (1.83–1.80)
Total reflections	568364
Unique reflections	81838
Multiplicity	6.9 (6.9)
Completeness (%)^a^	99.8 (99.2)
Mean *I*/σ(*I*)^a^	30.6 (2.9)
*R*_merge_ (%)^a,b^	9.6 (89.3)
Wilson *B*-factor (Å^2^)	20.3
**Refinement**	
Resolution range (Å)	43.90–1.80
Reflections	81813
Reflections (test set)	4099
*R*_work_ (%)	15.3
*R*_free_ (%)	15.5
No. of molecules in the asymmetric unit	2
No. of protein atoms/water molecules	6925/782
Average *B*-factor values of protein/water (Å^2^)	21.8/34.4
Ramachandran plot:	
favored/outliers (%)	97.6/0.0
Rotamer outliers (%)	0.0
RMSD bonds (Å)/angles (°)	0.004/0.75

^a^Values for the outermost resolution shell in parentheses.

^b^*R*_merge_ = Σ_*h*_ Σ_*i*_ |*I*(*h*)_*i*_ − 〈*I*(*h*)〉|/Σ_*h*_ Σ_*i*_ *I*(*h*)_*i*_, where *I*(*h*) is the observed intensity of reflection h, and 〈*I*(*h*)〉 is the average intensity obtained from multiple measurements.

**Figure 2:**
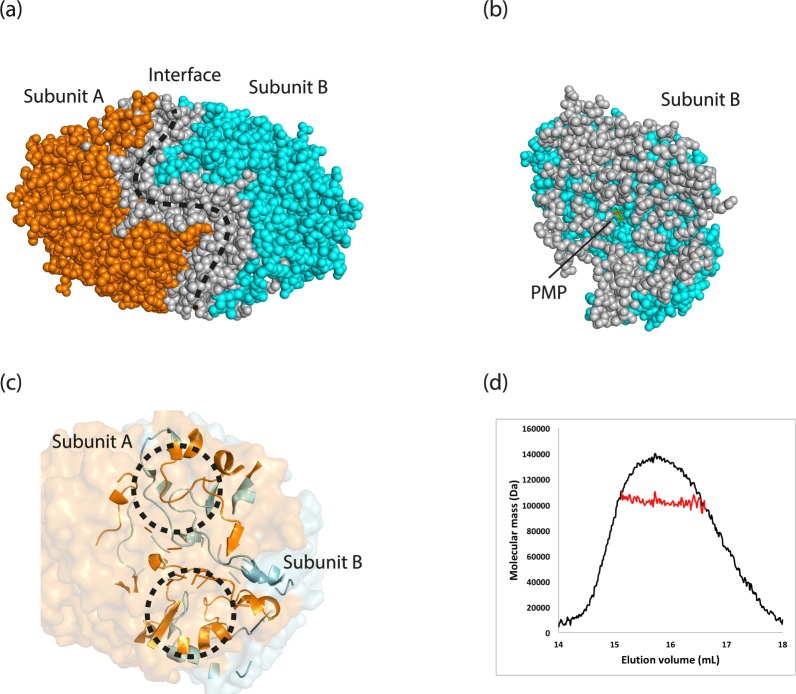
Dimer formation of Tr-ωTA. (**a**) Interactions between the two subunits of Tr-ωTA. Subunit A, B, and the interface are represented as orange, cyan, and grey spheres, respectively. (**b**) A cross section of subunit B containing PMP. PMP is depicted as sticks. (**c**) Loop insertion at the interface. The black dashed circles indicate inserted loops between the two subunits. The colour code is the same as in panel a. (**d**) SEC-MALS profile of Tr-ωTA. SEC-coupled MALS data (red) are plotted as elution volume and molecular mass distributions, and superimposed on the SEC chromatogram (black) at 280 nm.

The crystal structure of Tr-ωTA revealed that Tr-ωTA exists as a homodimer in the asymmetric unit. A plethora of residues are involved in interactions between each subunit (Fig. 2a,b). Several structured loops from the two subunits interact with each other at the interface, forming a tight loop insertion between the two subunits (Fig. 2c). These interactions indicated that Tr-ωTA is stable as a dimer form owing to the strong binding. A size exclusion chromatography-coupled multiangle light scattering (SEC-MALS) analysis also showed that a peak appeared at 15 ml retention volume, which indicated that Tr-ωTA exists as a dimer in solution (Fig. 2d). Moreover, a multimeric prediction using the PDBePISA server^28^ suggested that the most probable multimeric state of Tr-ωTA is a dimer in solution. These results coincided with the dimer form revealed by the crystal structure, strongly implying that Tr-ωTA carries out its biological function as a dimer.

Incorporating a cofactor into the active site has been shown to have an effect on conformational changes in partial regions of Vf-ωTA^25^. In our structure, PMP was observed only in subunit B. This finding raised the possibility that conformational differences might exist between the two subunits of Tr-ωTA. Accordingly, to investigate potential structural differences between the two subunits that form the dimer, their overall structures were compared (Supplementary Fig. S1). A structural superimposition analysis showed that the root-mean-square deviation (RMSD) value for 440 Cα atoms of each subunit was 0.71 Å. This RMSD value signified that the two subunits of Tr-ωTA are structurally almost identical to each other. As a result, although the PMP molecules were discriminatorily distributed between the subunits in the crystal, this did not cause any structural differences in the dimer form. It was thus inferred that the strong interactions between the two subunits could offset the structural dynamics induced by the absence of PMP.

**Figure 3:**
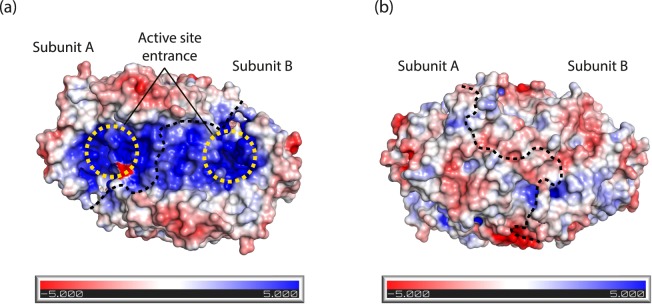
Surface electrostatic potential of Tr-ωTA. Surface electrostatic distribution is represented in two different orientations. The scale ranges from −5 kT/e (red) to 5 kT/e (blue). Electrostatic potential on the surface representing the active site entrances (**a**) and on the reverse side of panel a (**b**). The yellow dotted circles in panel a denote the active site entrances. The dashed curves indicate a boundary between the two subunits.

The surface electrostatic potential of Tr-ωTA was also assessed to investigate the spatial distribution of charged residues. Notably, we found that positively charged residues are dominantly distributed on the flat area including the entrance to the active site, whereas negatively charged residues are spread out on the peripheral “wall” structure surrounding the flat area (Fig. 3). However, the opposite side of the flat area did not exhibit any special features. This finding implied that the electric field generated in the vicinity of the active site may play an essential role in inducing the substrates into the active site (as discussed in further detail later in this report).

### Active site of Tr-ωTA

**Figure 4:**
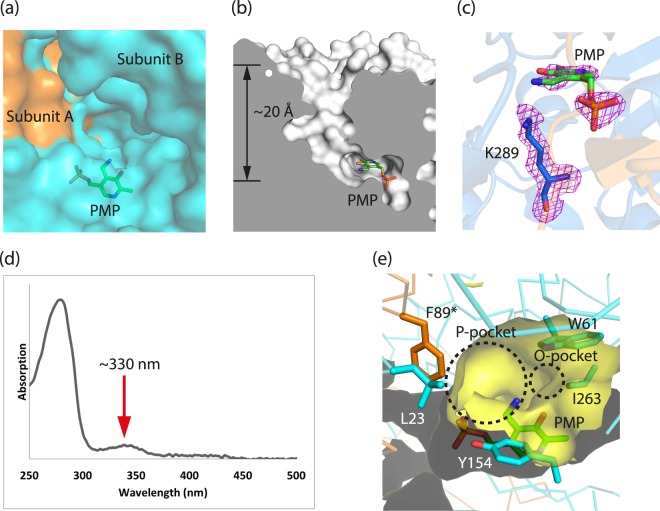
PMP-incorporated active site of Tr-ωTA. (**a**) Active site cavity of Tr-ωTA. The PMP cofactor is positioned in the vicinity of the interface of the two subunits. Some residues from subunit A are involved in the formation of the active site of subunit B. The apo enzyme and PMP are represented as surface and stick (green) modes, respectively. Subunit A and B are colored orange and cyan, respectively. (**b**) A cross section representing a funnel to the active site. The PMP cofactor is located in the bottom of the long funnel. Tr-ωTA is depicted as a surface (white) mode. (**c**) Omit map of PMP and Lys289. The omit map (*F*_O_–*F*_C_) is colored magenta and contoured at the 2.0 sigma level. (**d**) UV-Visible spectrum of Tr-ωTA. The red arrow indicates the peak position at approximately 330 nm. (**e**) The P- and O-pockets in the active site. The apo enzyme except for the active site (yellow surface and sticks) is represented in ribbon. Subunits A and B are colored orange and cyan, respectively. The asterisk of the F89 residue denotes a residue from the other subunit.

The active site of ωTAs is located at the interface between two subunits; moreover, some residues from one subunit are directly associated with the formation of the active site of the other subunit^1,2^. The entrance to the active site is also positioned near the interface. Another notable structural feature of ωTAs is the relatively deep funnel located between the entrance and the PLP-binding site^1,2^. These distinctive active site structural traits are conserved in Tr-ωTA (Fig. 4a,b).

The chemical modification state of PLP is important throughout the catalytic reaction of ωTAs because PLP plays an essential role as an amino group mediator^2,6^. The amino group transfer mechanism of ωTAs had been partially proposed on the basis of previously accumulated findings^2^. In the “rest period” of the catalysis, the C4′ atom of PLP covalently binds to the ε-amino group of an adjacent Lys residue, forming a Schiff base (the internal aldimine state). Subsequently, an amino group from a primary amine is substituted for the ε-amino group of the Lys residue, followed by the formation of PMP (the Michaelis complex state). In the Michaelis complex, consequently, PMP binds to neither the substrate nor the Lys residue. In this regard, determining whether a cofactor molecule in a crystal structure is PLP or PMP is significant because it provides decisive information to infer which step the structure corresponds to in the catalytic cycle.

In the crystal structure of Tr-ωTA, the cofactor molecule did not link to either the Lys289 residue or a possible substrate, suggesting that the cofactor was PMP (Fig. 4c). To further verify this conclusion, a UV-Visible spectrometric analysis of Tr-ωTA was also carried out to obtain the absorption spectrum of the cofactor. A previous study demonstrated that the PMP-bound form in a TA from *Thermoproteus uzoniensis* has an absorption peak at approximately 330 nm, whereas the absorption peak in the PLP-bound form appears in the proximity of 420 nm^29^. As shown in Fig. 4d, Tr-ωTA exhibited an absorption pattern at approximately 330 nm. This spectrometric result was in agreement with the PMP-absorption wavelength reported in the previous study^29^, confirming that the crystal structure of Tr-ωTA constituted the PMP-bound form.

The electron density of PMP is relatively poor as compared with that of K289 (Fig. 4c). Moreover, a series of omit maps of PMP at sequential sigma levels showed that the density signal of PMP is weak (Supplementary Fig. S2a–c), indicating that PMP exists at a lower molar ratio than the peptide component in the crystal. Additionally, to estimate the occupancy value of PMP, PMP-calculated 2*F*_O_–*F*_C_ and *F*_O_–*F*_C_ maps were generated with various occupancy values (0.83–0.25). We found that the PMP-calculated *F*_O_–*F*_C_ map shows the least electron density as both positive and negative maps at the 3 sigma level along with the clear 2*F*_O_–*F*_C_ map, when the occupancy value is set to 0.75 (Supplementary Fig. S2d,e). Consequently, the occupancy value of PMP in our structure is assumed to be approximately 0.75. As PMP is present only in subunit B in the crystal structure, our crystal therefore likely constituted a mixture of the apo and holo forms of Tr-ωTA. The scarcity of PMP in the crystal may result from loss during the purification and crystallization consequent to diffusion of PMP into the buffer solutions.

The Tr-ωTA structure also revealed that PMP is chemically linked to neighboring water molecules and residues (Supplementary Fig. S3). Specifically, the oxygen atoms in the phosphate group of PMP are coordinated to adjacent water molecules and residues such as Gly121, Ala122, and Thr323. The nitrogen atom on the PMP ring forms a hydrogen bond with Asp260. The hydroxyl group of the PMP also forms a hydrogen bond with a water molecule coordinated to Glu227 and Ala232, and the nitrogen atom linked to the C4′ atom binds to a water molecule as well. Overall, the PMP molecule is coordinated to the proximal residues via hydrogen bonds, part of which are mediated by water molecules. Accordingly, the hydrogen-bond network serves as a main factor underlying PMP coordination in the active site. It was also found that the carbon atoms on PMP form hydrophobic interactions with nearby hydrophobic residues such as Gly156, Val262, Ile263, and Phe322. Notably, Thr323 and Tyr324 from the other subunit contribute to the stabilization of the PMP position. This finding suggested that the dimer formation of Tr-ωTA is therefore indispensable to the catalytic activity.

The differently sized P- and O-pockets that comprise the ωTA active site^2,5,10^ are located in the proximity of the phosphate and the phenol group of PLP/PMP, respectively, and their relative sizes are dependent on the fold type and class of ωTAs^5,8^. In the case of Tr-ωTA, we found that the P-pocket is larger than the O-pocket (Fig. 4e). This implied that a bulkier functional group of (*S*)-enantioselective substrates would be oriented toward the P-pocket, whereas a smaller functional group is assigned to the O-pocket.

### Structural comparison with Vf-ωTA

**Figure 5:**
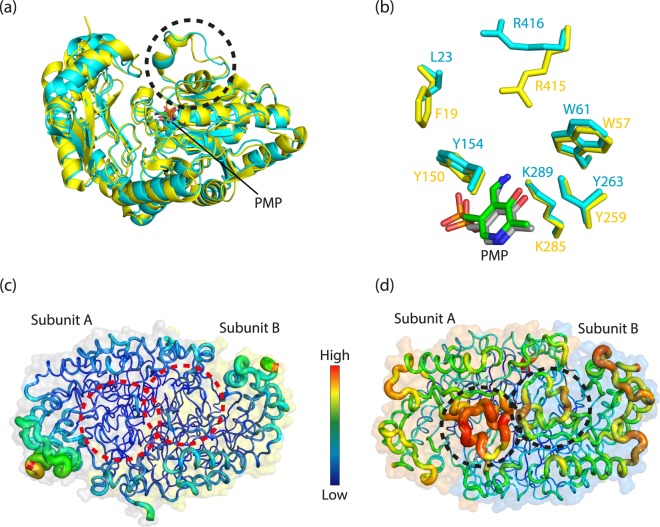
Structural comparison of Tr-ωTA with Vf-ωTA. (**a**) Overall structural comparison between Tr-ωTA and Vf-ωTA. Subunit B of Vf-ωTA is superimposed onto that of Tr-ωTA. The structures of Tr-ωTA and Vf-ωTA are colored cyan and yellow, respectively. The apo enzymes and PMP are shown in cartoon and stick representations, respectively. (**b**) Zoom-in view of the PMP cofactor in a different orientation. The PMP molecules and key residues of the active site are shown. The PMP cofactors of Tr-ωTA and Vf-ωTA are colored green and grey, respectively. *B*-factor distribution of Vf-ωTA (**c**) and Tr-ωλTA. (**d**) The two structures are depicted in putty representation and rainbow-colored from red to violet in *B*-factor value order. The dashed circles correspond to those in panel a.

The structure of Tr-ωTA was compared with that of Vf-ωTA (PDB ID: 4E3Q), which has a high sequence similarity (sequence identity = 41%)^27^. The structural comparison revealed that the two TAs are structurally almost identical to each other, sharing an overall architecture (Fig. 5a). The RMSD value for the two TAs (each of 440 Cα atoms) was 1.5 Å. The geometries of the active sites containing PMP were also in accordance (Fig. 5b), which suggested that Tr-ωTA may exhibit the same catalytic properties as Vf-ωTA.

Despite the structural and functional similarities, however, it was reported that Tr-ωTA exhibits an outstanding thermostability as compared with that of Vf-ωTA^27^. As protein mobility has been highlighted as an important factor contributing to temperature tolerance^30^, we analysed the *B*-factor distribution of the two TAs. This analysis showed that the structure of Vf-ωTA exhibits overall low *B*-factor values (Fig. 5c). In contrast, the loop covering the active site of Tr-ωTA exhibited high *B*-factor values (Fig. 5d), suggesting that this loop likely manifests intrinsic flexibility. This finding implied that the high mobility loop may play a key role in maintaining the structure of the active site by alleviating thermal perturbation caused by high temperature.

### Molecular docking simulations

Although the enantioselectivity of Tr-ωTA, a fold type I- and class III-ωTA, has been demonstrated^27^, the molecular mechanism has remained unclear. To obtain structural insight into (*S*)-enantioselective substrate recognition, we performed molecular docking simulations. Energetically favorable conformers of several compounds including *S* and *R* forms were investigated. Specifically, we selected (*S*)-enantioselective compounds reported as exhibiting relatively low and high activities in the previous study^27^; the (*R*)-enantiomers of the compounds with high activities were also prepared.

To more accurately run the docking simulations, some residues related to the active site were designated as flexible residues. The active site residues of (*S*)- enantioselective ωTAs have been identified on the basis of sequence alignment and docking simulation studies^7^. The putative active site residues of Tr-ωTA were also revealed by multiple sequence alignment with other ωTAs (Supplementary Fig. S4). For Tr-ωTA, Y154 along with L23 and F89 from the other subunit constitute the P-pocket components, whereas W61 and I263 are involved in the formation of the O-pocket. Additionally, K289 is assumed to play a crucial role in forming a Schiff base with PLP. An arginine residue corresponding to R416 has also been shown to be responsible for substrate binding^31^. Thus, these seven residues were set as flexible residues for the docking simulations. PMP in the structure was replaced with PLP in a Schiff base state for accurate docking simulations. The selected compounds were docked into the active site of Tr-ωTA under the same conditions.

**Figure 6:**
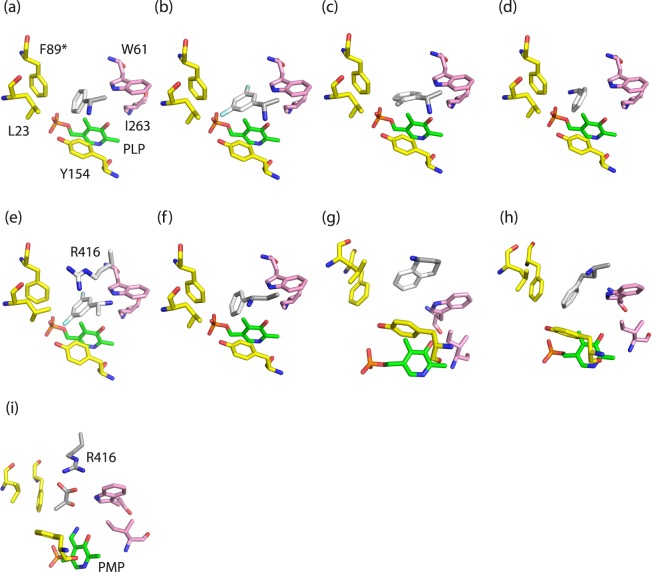
Predicted binding poses of the top scoring conformers. All the conformers, the active site residues, and the cofactor are shown in stick representation. The P-pocket is composed of L23, F89, and Y154, and colored yellow. The asterisk of F89 denotes a residue from the other subunit. The O-pocket consists of W61 and I263, and is colored pink. The cofactor and conformers are colored green and grey, respectively. The residue configurations in the active site are represented with the top scoring conformers for (*S*)-compound **1** (**a**) **2** (**b**) **3** (**c**) **4** (**d**) (*R*)-compound **2** (**e**) (*S*)-compound **5** (**f**) **6** (**g**) **7** (**h**) and pyruvate (**i**).

Each compound and its binding energy are listed in Supplementary Table S1. Notably, as the binding energy values of the compounds with the same formula are similar regardless of enantioselectivity, the docking products can be assessed only on the basis of their conformations. The four compounds with positive relative activities exhibited similar functional group orientations (Fig. 6a–d). Their bulky groups and short groups were oriented toward the P-pocket and O-pocket, respectively. In particular, the two fluorine atoms of compound **2** were shown to form hydrogen bonds with W61 and Y154 (Fig. 6b). A previous study demonstrated that a 1,3-proton transfer reaction mediated by the ε-amino group of the Lys key residue in the active site is a rate-determining step^32^. This reaction corresponds to the α-proton removal from an amino group donor (external aldimine) by the Lys residue and the subsequent α-proton transfer to the C4′ carbon atom of the cofactor (ketimine), which leads to the formation of the Michaelis complex via addition of a water molecule. Accordingly, considering that the spatial accession of the α-proton to the ε-amino group of the Lys residue is important for the general base catalysis, the binding modes of the substrate compounds notably for orientation and distance toward the Lys residue are assumed to be crucial for catalysis. In conclusion, these binding modes appeared to be appropriate as binding poses for substrate binding and catalysis. Moreover, these results coincided with the previous activity profiles for each compound^27^.

To investigate the substrate suitability of the (*R*)-enantiomer, the (*R*)-enantiomer for (*S*)-compound **2** was also prepared and subjected to a docking simulation. As shown in Fig. 6e, the orientations of its two functional groups were opposite to those of the (*S*)-enantiomer. In this binding pose, the amino group was placed away from the position of PLP. It also appeared that it was difficult for the small functional group to bind to the O-pocket. In addition, (*S*)-compounds with low relative activities showed inappropriate poses for the pockets and PLP (Fig. 6f–h). Thus, it was concluded that the binding of two functional groups to the two pockets and the orientation of an amino group depended on the size of the functional groups and the enantioselectivity of the compounds.

For class-III ωTAs, pyruvate can function as an amino group acceptor^1,2^. However, the structural mechanism at the molecular level is not fully understood. In the present study, we performed docking simulations of pyruvate to obtain structural insight into the transfer of an amino group to pyruvate. PMP was utilized in the structure as for the docking simulations. Notably, we found that the carboxylic acid of pyruvate interacts with the guanidino group of R416, resulting in the proper orientation of the ketone group toward PMP with a stable pose in the hydrophobic active site (Fig. 6i). From the viewpoint of thermodynamics, the entry of pyruvate into the active site of Tr-ωTA would cause an entropic decrease. Consequently, this would appear to result in an increase of free energy upon pyruvate binding. However, such a seemingly unfavorable phenomenon might be explained by the compensation of enthalpy. It is reasonable to assume that a hydrophilic substrate such as pyruvate would be able to maintain a thermodynamically stable state by increasing the number of binding points to adjacent residues, thereby decreasing enthalpy under thermodynamically unfavorable circumstances such as within the hydrophobic active site.

### Amino group transfer mechanism of Tr-ωTA

**Figure 7:**
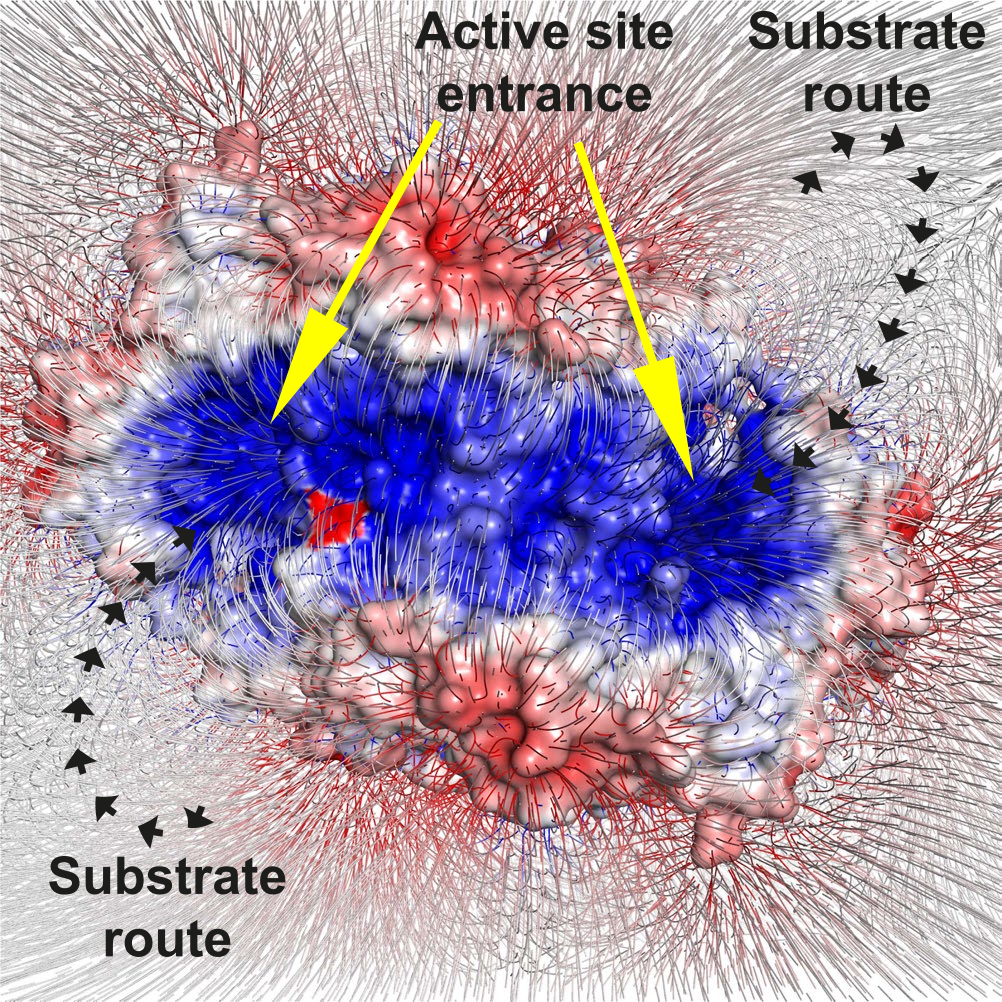
Electric field generation of Tr-ωTA. Surface electrostatic potential is represented in the same method as in Fig. 3. The electric field map grad is contoured at the −0.5 sigma level.

Two substrates are necessary for the completion of the transamination reaction by TAs using PLP as a cofactor. Notably, both PLP and pyruvate have strong negative charges, which supports the likelihood of a mechanism by which the charged molecules are induced into the active site of Tr-ωTA. Our surface electrostatic potential analysis revealed that positively charged residues are distributed in the proximity of the active site (Fig. 3), thereby generating a potent electric field (Fig. 7). This electric field may play a pivotal role in attracting PLP and pyruvate into the active site. In addition, we also found that hydrophobic residues are dominantly distributed in the interior as well as at the entrance of the active site (Supplementary Fig. S5). The distribution of these hydrophobic residues in the active site may constitute a useful strategy for favorably disposing PLP and the substrates in the active site by obstructing inappropriate interactions with the charged molecules.

This assumption is supported by a previously published example regarding acetylcholinesterase, wherein this enzyme was reported to exhibit a strong electrostatic dipole^33^. This study raised the possibility that the electric field induces the charged substrate into the active site of acetylcholinesterase and that aromatic residues in the active site are associated with the formation of a proper binding site. Another study demonstrated that electrostatic properties near the binding sites of enzymes such as superoxide dismutase, acetylcholinesterase, and barnase are associated with electrostatic steering for the substrate binding^34^. This study showed that introduction of charged residues near the active site increased enzyme-substrate association rates, implying electrostatic enhancement. The results were reproduced using Brownian dynamics simulations based on the finite-difference linearized Poisson-Boltzmann equation. In turn, Tr-ωTA may constitute another example demonstrating the importance of an electric field with respect to transporting a substrate into its active site.

**Figure 8:**
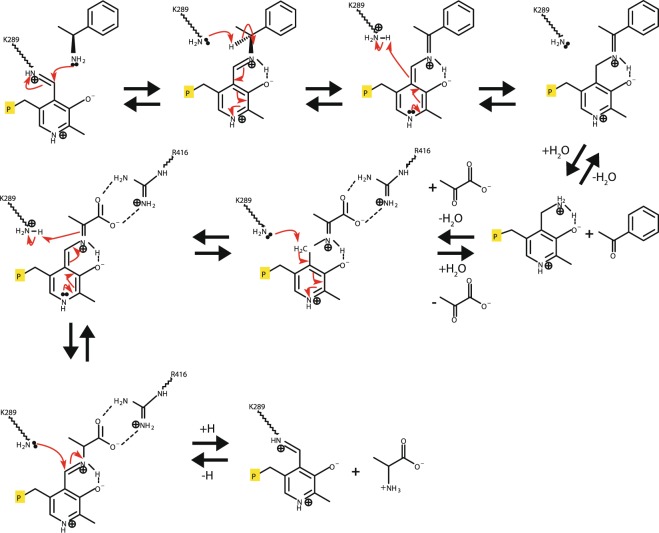
Proposed catalysis mechanism for Tr-ωTA. (*S*)-α-MBA and pyruvate are used as model substrates for amino group donation and acceptance, respectively. The red arrows denote the attack directions of a pair of electrons. The yellow-boxed P letter symbolizes the phosphate group of the PLP/PMP cofactor.

TAs including Tr-ωTA exhibit the enzymatic property to sequentially recognize two different substrates at the same active site. Accordingly, the transamination reaction of Tr-ωTA comprises two consecutive processes: amino group donation and acceptance (Fig. 8). Herein, we propose a comprehensive transamination mechanism including these two processes. As the initial step, a nucleophilic attack from the first substrate as an amino group donor allows the Lys289 residue to depart from the bound PLP molecule, forming a new Schiff base between the substrate and PLP. A straightforward base abstraction of the α-proton by the Lys289 residue causes electronic rearrangement in the external aldimine step. After the acceptance of a proton from the Lys289 residue, a hydrolysis reaction occurs, producing the Michaelis complex and a ketone compound. Then, the second process is initiated through the use of pyruvate as an amino group acceptor. Interacting with the R416 residue, pyruvate forms a ketimine with PMP. The second proton acceptance from the Lys289 residue occurs along with electron rearrangement, resulting in the second external aldimine. Finally, imination is attained between the Lys289 residue and PLP, recovering the initial internal aldimine.

In summary, we determined the crystal structure of Tr-ωTA specific to (*S*)-enantiomers. Surface electrostatic potential analysis revealed that Tr-ωTA likely exploits an electric field to induce its cofactor PLP and substrates into the active site. Based on the molecular docking simulations, we concluded that the orientations of the functional groups and amino group at the chiral center of an amino group donor comprise determining factors for the (*S*)-enantioselectivity of Tr-ωTA. In addition, pyruvate as an amino group acceptor was assumed to bind to the R416 residue, attaining binding stability at the active site. To more specifically understand the catalytic mechanism of Tr-ωTA, it is necessary to elucidate the stepwise intermediate states on the basis of structural information. Thus, future work should determine the respective complex structures by crystallizing the substrate-bound forms of Tr-ωTA. Nevertheless, our results lay the groundwork for a more comprehensive understanding of the transamination mechanism.

## Methods

### Cloning, overexpression, and purification

The pET24a(+) expression vector for Tr-ωTA was constructed. The plasmid was delivered into *Escherichia coli* BL21(DE3) for transformation. A single colony was selected and cultured in lysogeny broth medium containing 50 μg/ml kanamycin at 37 °C overnight. The resulting cells were cultured on a large scale at 37 °C until the optical density value at 600 nm reached approximately 0.6. Overexpression of the gene was induced with 0.5 mM isopropyl β-D-1-thiogalactopyranoside and the cells were further cultured at 20 °C for 18 h. The cultured cells were harvested by centrifugation, washed with buffer A [20 mM Tris-HCl (pH 8.0), 500 mM NaCl, and 20 mM imidazole], flash-frozen with liquid N_2_, and stored at −80 °C until use.

The cell pellet was resuspended with buffer A supplemented with phenylmethanesulfonyl fluoride (Sigma-Aldrich) as a serine protease inhibitor and lysed by sonication on ice using 30-s bursts with a 1-min time interval between each burst. The cell debris was removed by centrifugation at 10,000 g for 30 min at 4 °C. The supernatant was mixed with Ni-nitrilotriacetic acid resins by gentle agitation overnight. The mixture was loaded onto a gravity-flow column pre-equilibrated with buffer A. The column was washed with buffer B [20 mM Tris-HCl (pH 8.0), 500 mM NaCl, and 60 mM imidazole] twice. Then, the Tr-ωTA protein was eluted with buffer C [20 mM Tris-HCl (pH 8.0), 500 mM NaCl, and 250 mM imidazole]. The eluate was loaded onto a Superdex 200 Increase 10/300 GL 24 ml column (GE Healthcare) pre-equilibrated with buffer D [20 mM Tris-HCl (pH 8.0), 150 mM NaCl]. SEC purification was performed using an ÄKTA explorer system (GE Healthcare). Protein fractions were harvested, concentrated to 10 mg/ml using a centrifugal 0.2-μm filter, flash-frozen in liquid N_2_, and stored at −80 °C until use for crystallization experiments. All of the purification fractions were analyzed by SDS-PAGE.

### Crystallization and data collection

The initial crystallization conditions were screened using commercial screening kits such as Wizard I and II (Hampton Research). Crystals were initially obtained from a buffer condition consisting of 1 M sodium citrate tribasic and 0.1 M imidazole/HCl (pH 8.0). The crystallization condition was optimized by modulating buffer concentration and pH, and supplementing an additive. For droplet preparation, 1 μl of protein solution was mixed with an equal volume of reservoir solution. Each droplet was equilibrated against 400 μl of the mother liquor using the hanging drop vapor diffusion method at 20 °C. Diffraction-quality crystals appeared in 10 days under a buffer condition of 0.9 M sodium citrate tribasic, 0.1 M imidazole/HCl (pH 7.4), and 0.1 M betaine-HCl. The crystals were soaked in a cryoprotectant solution comprising the reservoir solution supplemented with 15% (v/v) glycerol. Then, the crystals were mounted onto a goniometer head and flash-cooled in a N_2_ stream at −178 °C. X-ray diffraction data for Tr-ωTA were collected at the PAL 5C beamline (Pohang, Korea). Indexing, integrating, and scaling of the diffraction data were processed using HKL2000 software^35^.

### Structure determination and refinement

The phase of Tr-ωTA was determined by molecular replacement using Phaser-MR^36^ in Phenix^37^. The structure of Vf-ωTA (PDB ID: 4E3Q) was used as a search model. The initial model of Tr-ωTA was automatically built with AutoBuild^38^ in Phenix, and a portion was manually built using Coot^39^. Iterative cycles of refinement were run using phenix. refine^40^ in Phenix. The model refinement was finished when *R*_work_ and *R*_free_ values reached 15.3% and 15.5%, respectively. Validation for the final model was performed using MolProbity^41^.

Multiple sequence alignment was performed and displayed using Clustal Omega^42^ and ESPript 3.0 (http://espript.ibcp.fr)^43^, respectively. All the structural figures shown in this study were prepared using Pymol^44^.

### Molecular docking simulations

Substrate candidates for Tr-ωTA were prepared using Avogadro^45^. All the structures were energetically minimized. Molecular docking simulations were performed using Autodock Vina^46^, a program package for molecular docking. AutoDockTools 1.5.6^47^ was used to generate the pdbqt files of Tr-ωTA and the compounds. The active site-forming residues such as L23, W61, Y154, I263, K289 from subunit B, and F89 from subunit A were designated as flexible residues and the rest residues were set to rigid residues during the docking simulations. The size and spacing of a grid box including the flexible residues were set to 20 Å × 20 Å × 20 Å and 1 Å, respectively. Other parameters were assigned default values. A total of 15 different conformers per docking compound were generated. The docking results were ranked using an energy scoring function.

### Multiangle light scattering analysis

A SEC-MALS analysis was conducted to measure the absolute molecular weight of Tr-ωTA in solution. The protein solution was loaded onto a Superdex 200 10/300 GL 24 ml column pre-equilibrated with a buffer comprising 20 mM Tris-HCl (pH 8.0) and 500 mM NaCl. The mobile phase was pumped at a flow rate of 0.4 ml/min at room temperature. A DAWN-treos MALS detector (Wyatt Technology) was used in conjunction with an ÄKTA explorer system. BSA was used as a reference. Data were assessed using ASTRA software provided by Wyatt Technology.

### UV-Visible spectroscopic analysis

A UV-Visible absorption spectroscopic analysis for Tr-ωTA was carried out using NanoPhotometer NP80 spectrophotometry (IMPEL) to specify a cofactor in Tr-ωTA. The light was generated using a xenon flash lamp with a bandwidth of 1.8 nm. An absorption spectrum was obtained from a wavelength range of 200–900 nm. Wavelengths for peak absorbance were analyzed to identify corresponding molecules.

## Supplementary information

Supple_figure

## Data Availability

The atomic coordinates and structure factors for Tr-ωTA have been deposited in the Protein Data Bank (http://www.rcbs.org) under accession number 6IO1.
