# Supplementary material for: Structural basis of substrate recognition by a novel thermostable (S)-enantioselective ω-transaminase from Thermomicrobium roseum
Source: Sci Rep. 2019 May 6;9:6958. doi: 10.1038/s41598-019-43490-2 (PMC6502798; doi:10.1038/s41598-019-43490-2)
Supplement: Supplementary file 1 — Supple_figure [file 41598_2019_43490_MOESM1_ESM.docx]

Supplementary Information

**Structural basis of substrate recognition by a novel thermostable (*S*)-enantioselective ω-transaminase from *Thermomicrobium* *roseum***

Sunghark Kwon^a^, Jun Hyuck Lee^b^, Chang Min Kim^a^, Hyunseok Jang^a^, Hyungdon Yun^c^, Ju-Hong Jeon^d^, Insuk So^d^, and Hyun Ho Park^a^*

^a^College of Pharmacy, Chung-Ang University, Dongjak-gu, Seoul 156-756, Republic of Korea

^b^Unit of Polar Genomics, Korea Polar Research Institute, Incheon 21990, Republic of Korea

^c^Department of Systems Biotechnology, Konkuk University, Seoul 05029, Republic of Korea

^d^Department of Physiology and Biomedical Sciences, Institute of Human-Environment Interface Biology, Seoul National University College of Medicine, Jongno-gu, Seoul 03080, Republic of Korea

^*^To whom correspondence should be addressed: Hyun Ho Park, College of Pharmacy, Chung-Ang University, Dongjak-gu, Seoul 156-756, Republic of Korea, E-mail: xrayleox@cau.ac.kr


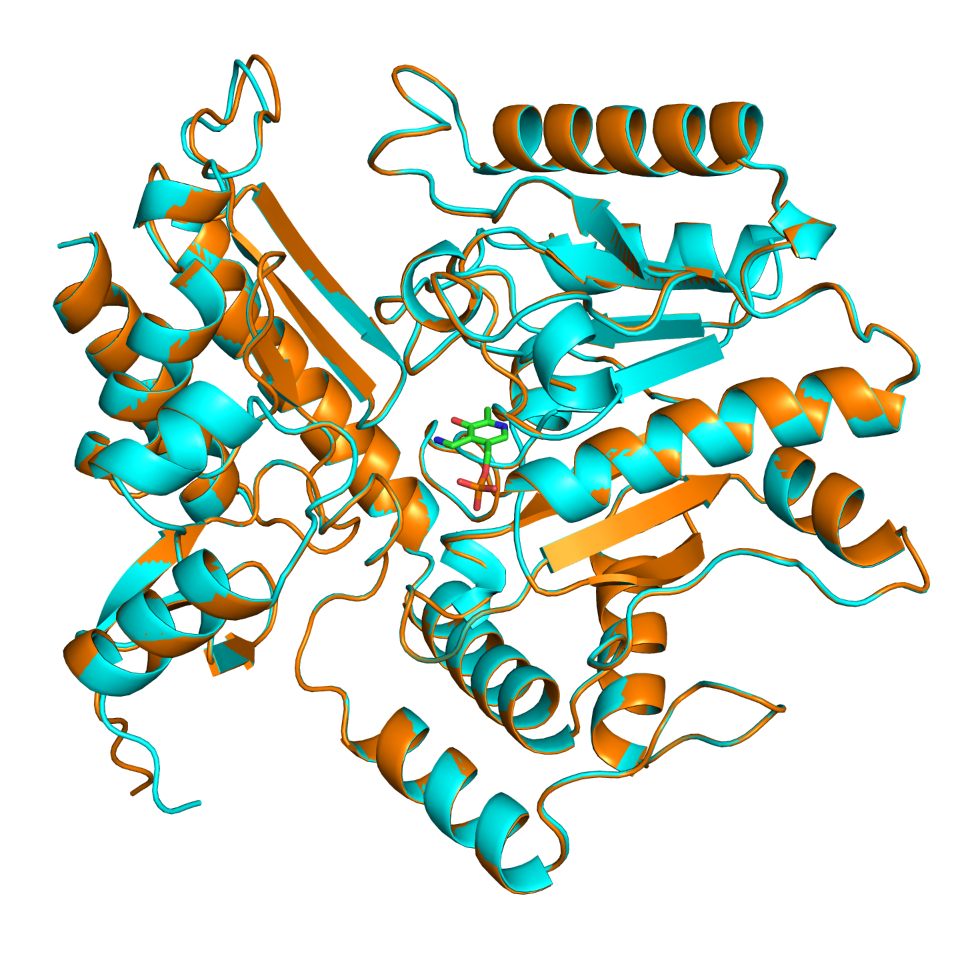


**Figure S1.** Structural comparison between the two subunits of Tr-ωTA. The two subunits are superimposed onto each other. Subunits A and B are coloured cyan and orange, respectively. The apo enzymes and PMP in subunit B are depicted in cartoon and stick representation, respectively.

**
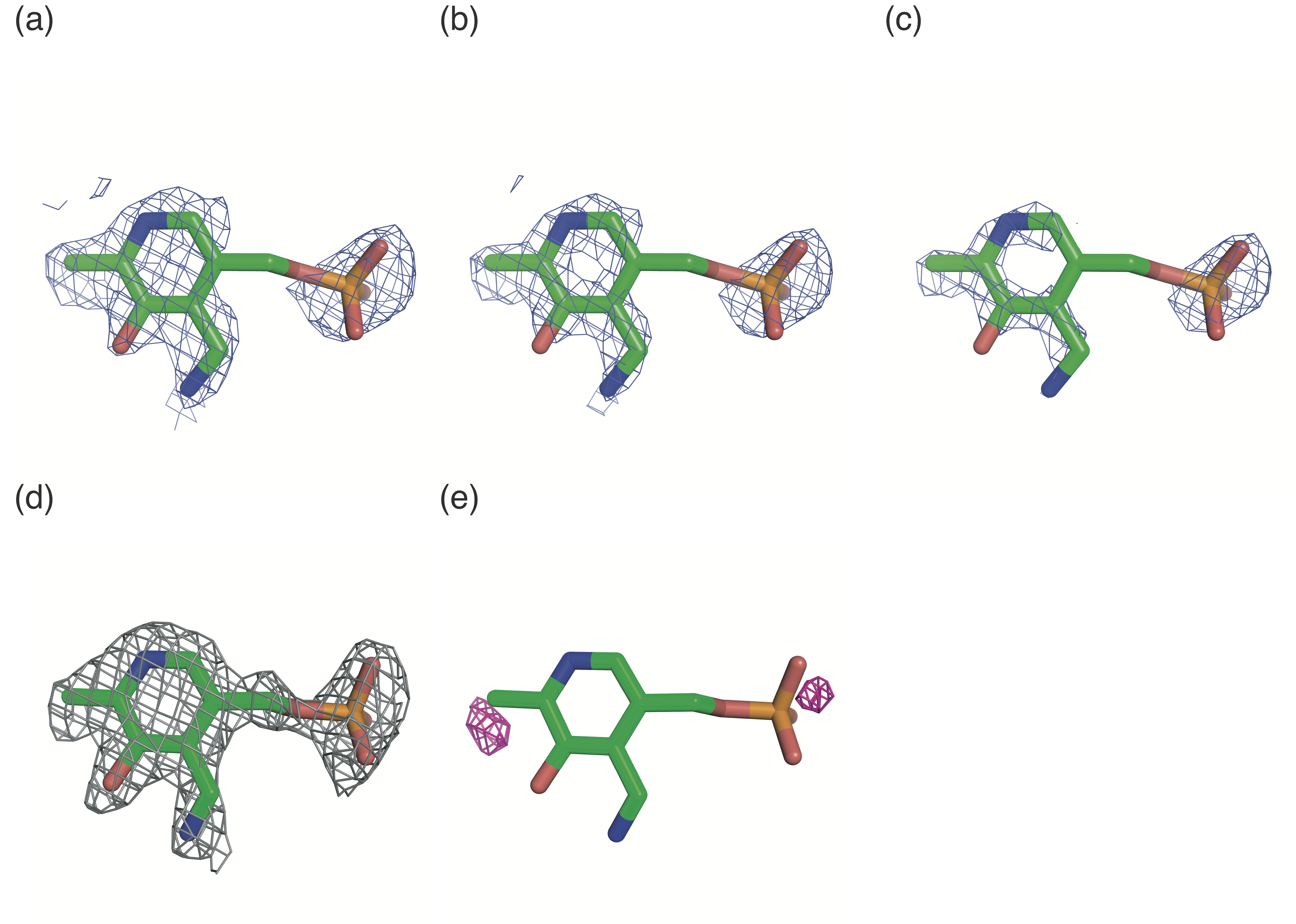
**

**Figure S2.** PMP omit maps (*F*_O_-*F*_C_) and PMP-calculated 2*F*_O_-*F*_C_ and *F*_O_-*F*_C_ maps. The omit maps are contoured at the 1.5 (a), 2.0 (b), and 2.5 (c) sigma levels. The PMP-calculated 2*F*_O_-*F*_C_ map (d) and *F*_O_-*F*_C_ map (e) are contoured at the 1.0 and 3.0 sigma levels, respectively.

**
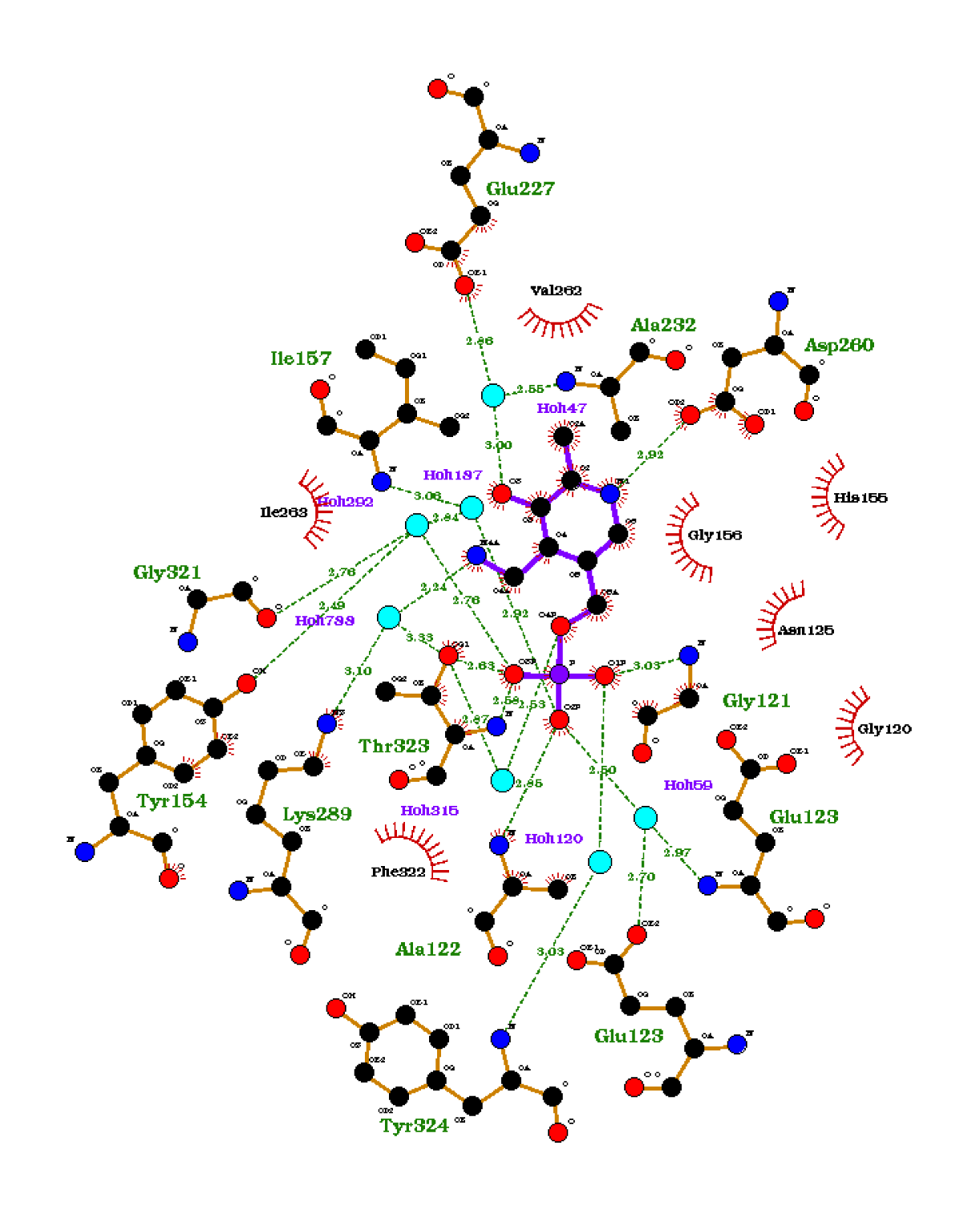
**

**Figure S3.** Diagram for PMP interactions in the active site. PMP interacts with water molecules and neighbouring residues.


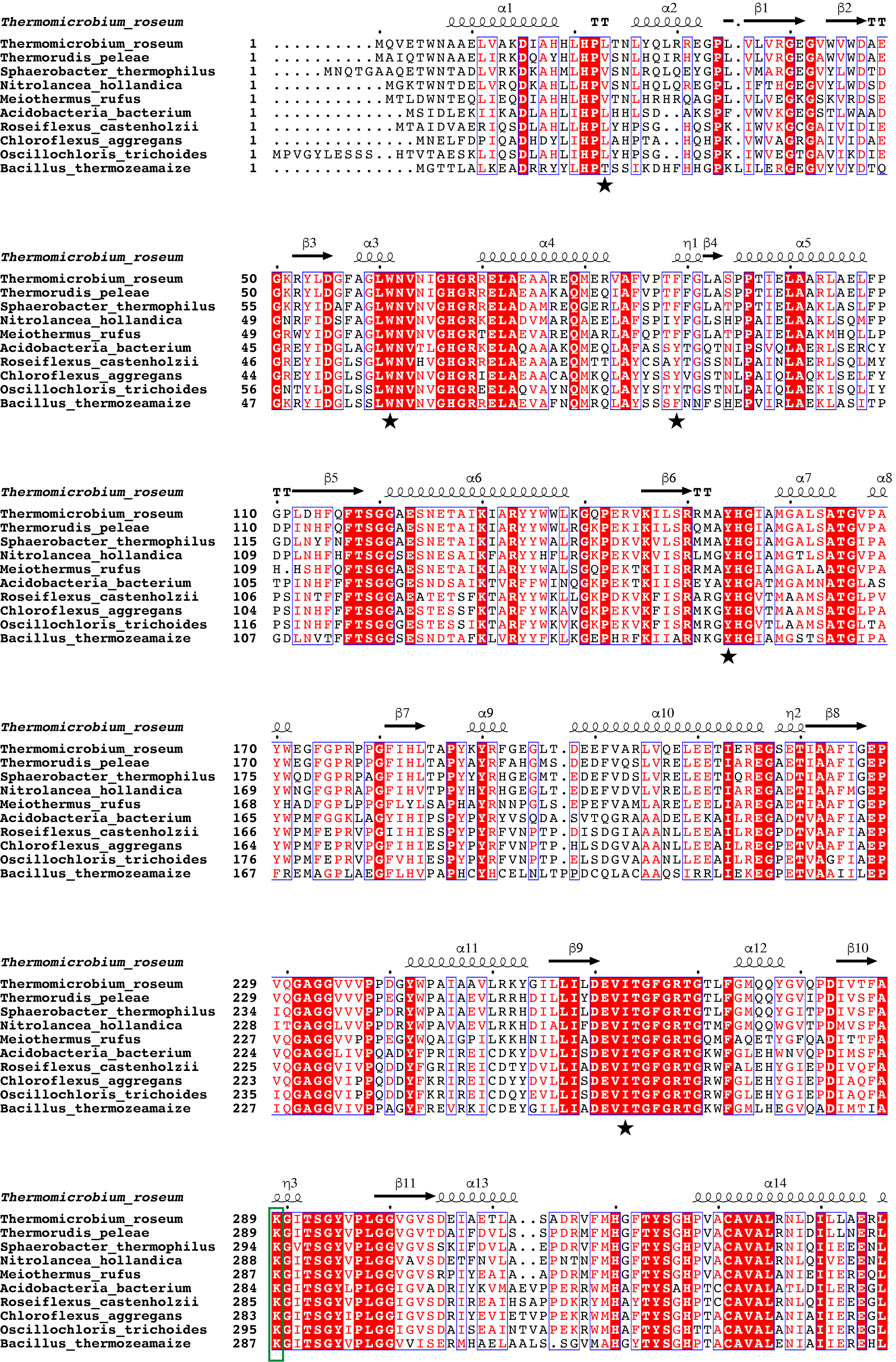


**
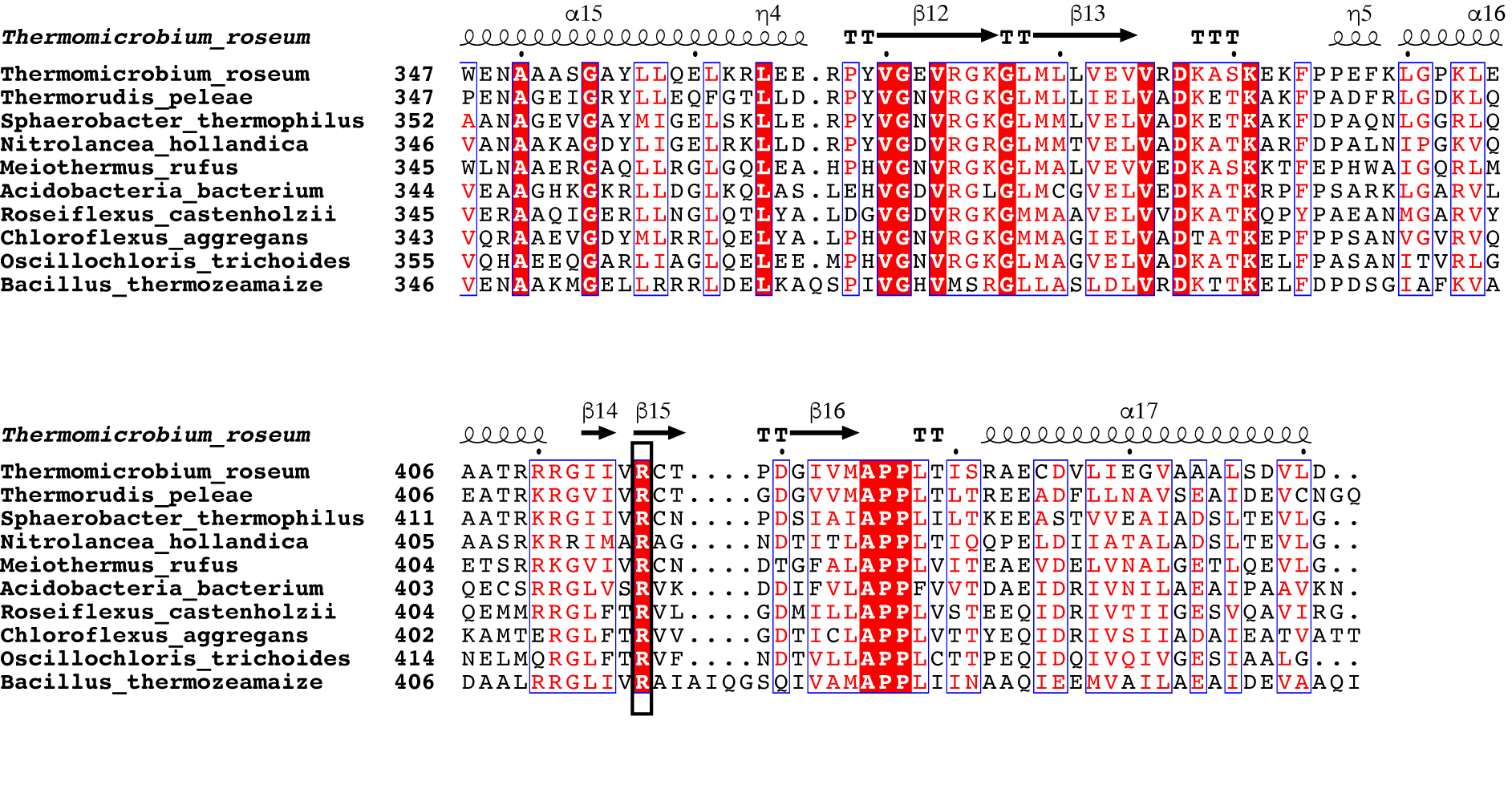
**

**Figure S4.** Multiple sequence alignment between Tr-ωTA and other ωTAs. The sequences are derived from *T. roseum*, *Thermorudis peleae*, *Sphaerobacter thermophilus*, *Nitrolancea hollandica*, *Meiothermus rufus*, *Acidobacteria bacterium*, *Roseiflexus castenholzii*, *Chloroflexus aggregans*, *Oscillochloris trichoides,* and *Bacillus thermozeamaize*. Completely identical residues are shaded in red, and similar residues are shown with red letters. Secondary structure elements of Tr-ωTA are denoted above the sequence, where TT symbolises a β-turn. The asterisks indicate residues forming the pockets in the active site. The green box marks lysine residues bound to PLP. The black box indicates arginine residues that probably interact with an amino group acceptor such as pyruvate or α-ketoglutarate.

**
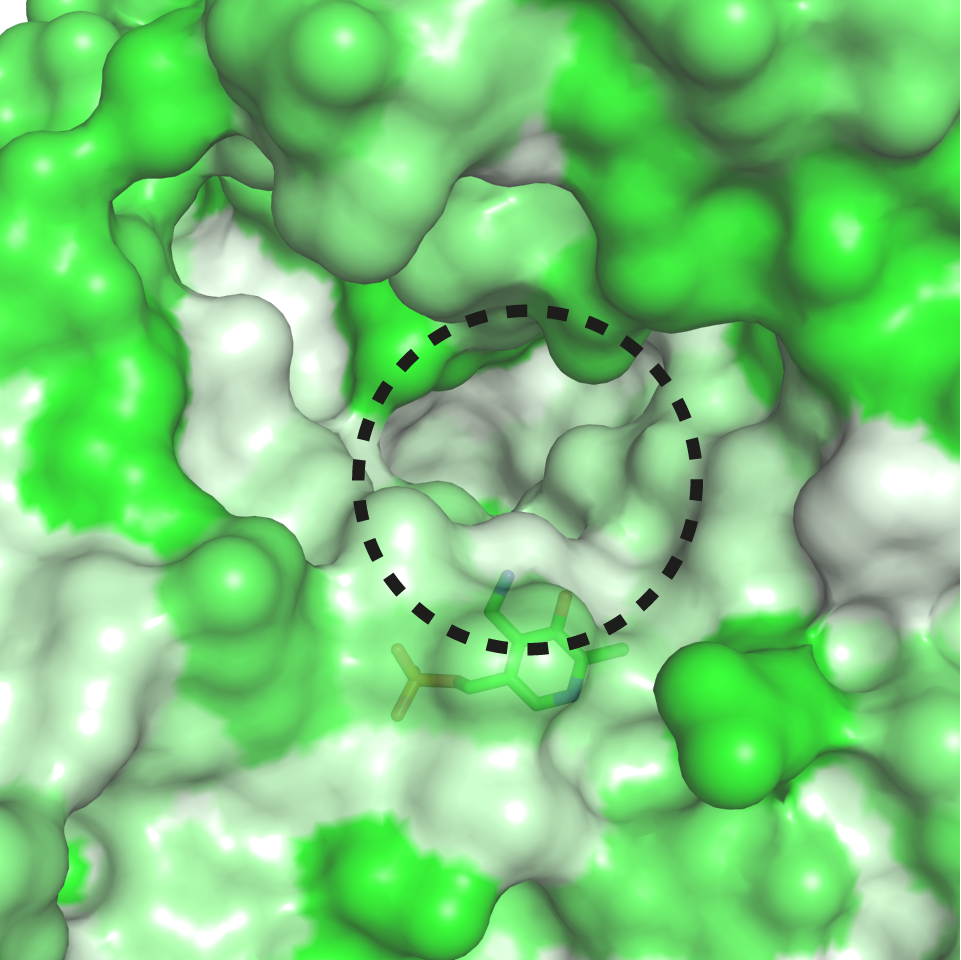
**

**Figure S5.** Surface hydrophobic analysis of the active site of Tr-ωTA. The degree of hydrophobicity increases from green to white. The dashed circle indicates the entrance and cavity of the active site.

Table S1. Compounds prepared for molecular docking simulations and their binding energy values

| # | Compound | Configuration | Binding energy (kcal/mol) |
| --- | --- | --- | --- |
| **1** | 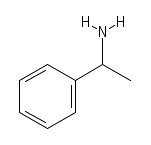 | (*S*) | -5.6 |
| **2** | 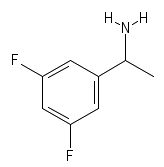 | (*S*) | -6.4 |
| **3** | 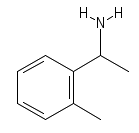 | (*S*) | -5.9 |
| **4** | 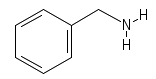 | (*S*) | -5.6 |
| **5** | 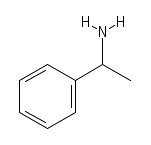 | (*R*) | -5.5 |
| **6** | 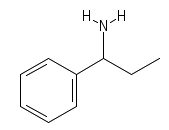 | (*S*) | -5.9 |
| **7** | 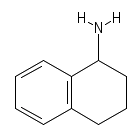 | (*S*) | -6.7 |
| **8** | 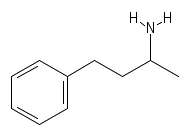 | (*S*) | -6.3 |
| **9** | 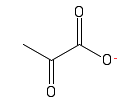 | (*S*) | -4.1 |
